# Mental disorder or conscious disturbance in epidermal growth factor receptor-tyrosine kinase inhibitor treatment of advanced lung adenocarcinoma

**DOI:** 10.17179/excli2019-1964

**Published:** 2020-02-28

**Authors:** Jing Zhu, Rui Zhou, Heng Xiao

**Affiliations:** 1 Department of Oncology, The First Affiliated Hospital of Chongqing Medical University, Chongqing, China; 2 Department of Hepatobiliary Surgery, The First Affiliated Hospital of Chongqing Medical University, Chongqing, China

**Keywords:** advanced lung adenocarcinoma, conscious disturbance, epidermal growth factor receptor-tyrosine kinase inhibitor, mental disorder

## Abstract

Epidermal growth factor receptor-tyrosine kinase inhibitors (EGFR-TKIs) are currently recommended by international guidelines as first-line treatment in patients with advanced EGFR-mutant non-small-cell lung cancer. With the availability of drugs, more and more patients choose EGFR-TKI treatment. However, pharmaceutical drugs used in clinical practice have side effects, such as diarrhea, paronychia, and hepatotoxicity. Mental or conscious disturbance has never been reported before. In our clinical center, we found that several patients with advanced lung adenocarcinoma developed a mental disorder or conscious disturbance after EGFR-TKI treatment. This situation has not previously been reported. We conducted a retrospective study of patients with advanced lung adenocarcinoma treated with EGFR-TKI who showed a mental disorder or conscious disturbance. We reported five cases of lung adenocarcinoma who developed a mental disorder or conscious disturbance after treatment with EGFR-TKI. The main clinical symptoms of these patients were sluggishness, memory deterioration, cognitive disorder, and even hallucination. Brain magnetic resonance imaging showed increased ischemic foci and lacunar infarction, worse encephalatrophy, and demyelination after EGFR-TKI therapy. These psychiatric symptoms did not improve but worsened after taking antipsychotic drugs, suggesting that they were irreversible. The neuropsychiatric symptoms in EGFR-TKI treatment must be considered, and the underlying reason warrants further study.

## Introduction

Lung cancer is the leading cause of cancer deaths globally. The most common subtype of lung cancer is non-small-cell lung cancer (NSCLC), accounting for approximately 80 % of all lung cancer cases. Most patients with NSCLC are diagnosed at advanced stages (IIIb and IV) of the disease, and patients with advanced NSCLC generally have a poor prognosis, with a median survival of 8-10 months (Polanski et al, 2016[16]). Epidermal growth factor receptor (EGFR)-mutant NSCLC is a typical example of “oncogene addiction” that can be treated by molecular agents that specifically target the constitutive activation of EGFR (Han et al., 2012[8]; Inoue et al., 2013[11]; Mok et al., 2009[14]). Since the discovery of EGFR-tyrosine kinase inhibitors (TKIs) that inhibit the activation of the intracellular domain of EGFR, substantial improvement in EGFR-mutant NSCLC has been achieved. EGFR-TKIs are currently recommended by international guidelines as first-line treatment in patients with advanced EGFR-mutant NSCLC (Hanna et al., 2017[9][10]; Planchard et al., 2018[15]; Reck et al., 2014[17]). EGFR-TKIs represent the standard of care for NSCLC patients harboring EGFR mutations. With the availability of drugs, more and more patients choose EGFR-TKI treatment. However, side effects of pharmaceutical drugs were observed in clinical practice. 

Adverse events of EGFR-TKIs include diarrhea, stomatitis and mucositis, paronychia, cutaneous adverse events, hepatotoxicity, interstitial lung disease, and so on (Biswas et al., 2017[5]). Mental or conscious disturbance has never been reported before. In our clinical center, several patients with advanced lung adenocarcinoma suffered a mental disorder or conscious disturbance after EGFR-TKI treatment. Hence, this article reports for the first time a series of clinical cases of a mental disorder or consciousness disturbance in the treatment of advanced lung adenocarcinoma with EGFR-TKIs in our center. 

## Case Presentation

### Case 1

**Figure 1:**
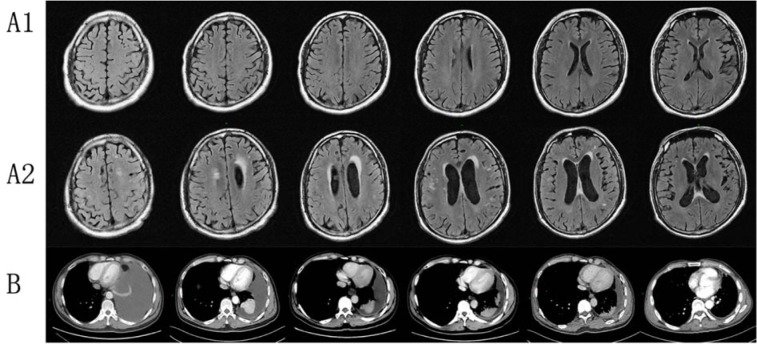
Clinical images of Case 1. (A1) Brain MRI with contrast of brain for patient 1 displaying normal status before EGFR-TKI therapy; (A2) Brain MRI showed increased ischemic foci and lacunar infarction, serious encephalatrophy, and demyelination after EGFR-TKI therapy; (B) Chest CT with contrast of patient 1 showed gradually decreasing pleural effusion in EGFR-TKI treatment.

A 44-year-old male (Figure 1(Fig. 1)) initially presented with cough and expectoration repeatedly in March 2016. Further image showed left massive pleural effusion and ipsilateral partial compressive atelectasis. Adenocarcinoma was detected by pleural fluid cytology and bronchial lavage fluid. Electroconvulsive therapy (ECT) and magnetic resonance imaging (MRI) suggested bone metastases. Brain MRI enhancement scanning showed no obvious abnormality, genetic testing revealed EGFR 19-del mutation, and EML4-ALK and ROS-1 were negative. The patient received combination chemotherapy (pemetrexed and nedaplatin), but chest CT examination demonstrated increased pleural fluid. Thus, the patient started targeted therapy (gefitinib, 250 mg, once a day) in April 2016. Periodic reviews showed gradually decreasing pleural fluid. After 9 months, reexamination suggested stable disease. After suspicion of brain metastasis, the patient switched from gefitinib to erlotinib (150 mg, once a day) by himself in January 2017. Periodic reviews suggest stable disease. However, the patient suffered from memory deterioration, sluggishness, hallucination, and persecutory delusion 3 months after targeted therapy. Cerebral MRI enhancement scanning showed increased ischemic foci and lacunar infarction, worse encephalatrophy, and demyelination. After consultation with radiologists, a psychiatrist, and a neurologist, metastatic tumor in brain was not considered, and olanzapine was administered. However, no obvious improvement was noted. Finally, this patient died in March 2018 outside the hospital with an unknown cause of death.

### Case 2 

**Figure 2:**
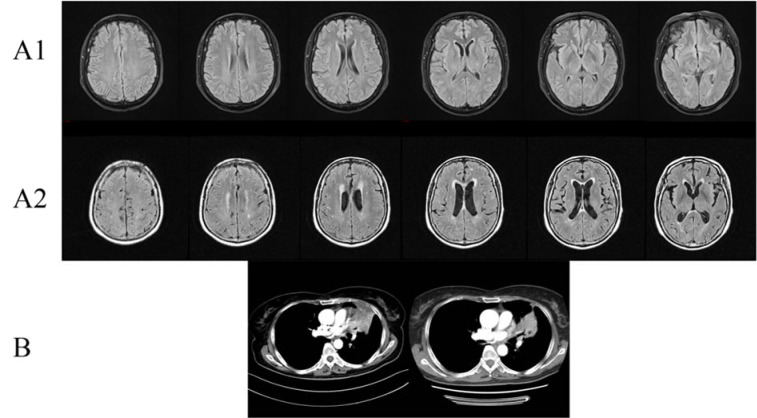
Clinical images of Case 2. (A1) Brain MRI with contrast showed suspicious reinforcement in the epencephalon and some ischemic areas in the frontal, parietal, and occipital lobes before EGFR-TKI therapy; (A2) Brain MRI showed multiple ischemic foci and lacunar infarction, encephalatrophy, and demyelination in white matter after EGFR-TKI therapy; (B) Chest CT with contrast of patient 2 showed partial response in chest lesions after EGFR-TKI treatment.

A 62-year-old female (Figure 2(Fig. 2)) initially presented with low back pain in February 2017. Further imaging demonstrated bone metastasis from lung cancer. Pathological examination indicated lung adenocarcinoma. Cerebral MRI enhancement scanning showed suspicious reinforcement in the epencephalon and some ischemic areas in the frontal, parietal, and occipital lobes. Genetic testing showed that EGFR, EML4-ALK, and ROS-1 were negative. After two cycles of chemotherapy (taxol and nedaplatin), reexamination showed the disease progressed. The patient started targeted therapy (gefitinib, 250 mg, once a day) by herself in May 2017. After 3 months, reexamination showed smaller pulmonary lesions and steady osseous lesions. Moreover, the patient suffered from difficulty in speaking, retardation, hypomnesis, and unstable gait. Cerebral MRI enhancement scanning showed multiple ischemic foci and lacunar infarction, encephalatrophy, and demyelination in white matter. The patient stopped targeted therapy, but these symptoms worsened and went into a coma in October 2017. The patient died after a brief period of time in coma.

### Case 3

A 62-year-old male initially presented with cough, sputum, and blood-stained sputum. After a medical examination, the patient was diagnosed with lung cancer and received right upper lobectomy and lymphadenectomy, Postoperative pathology suggested <the upper lobe of the right lung> invasive adenocarcinoma. However, PET-CT examination showed adrenal gland and bone metastasis. The patient received six cycles of chemotherapy (taxol and cisplatin) until the disease progressed. Then, the patient refused chemotherapy and blindly took the Indian version of gefitinib in November 2015. Reexamination showed disease progression in adrenal gland lesion. After 7 months, the patient suffered from sluggishness, memory deterioration, and cognitive disorder (e.g., did not know how to open the door when standing by the door, could not use chopsticks when eating, did not know how to unbutton his pants when urinating). However, this patient refused cerebral MRI enhancement scanning or further examination and died after best supportive care in March 2018. 

### Case 4

**Figure 3:**
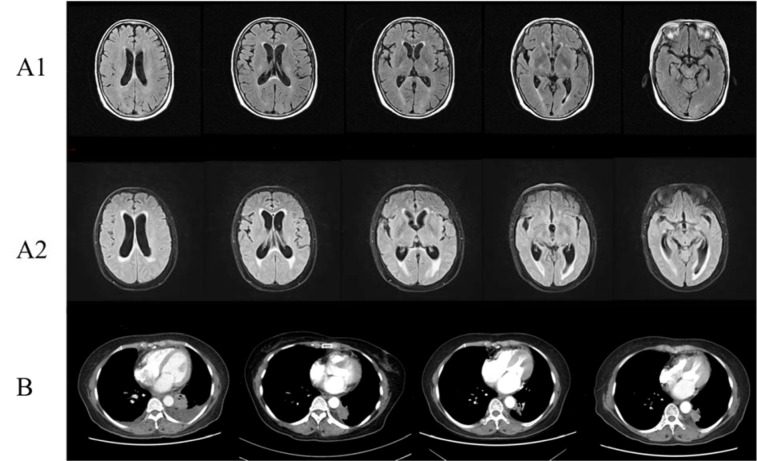
Clinical images of Case 4. (A1) Brain MRI showed some ischemic foci in the frontal lobe and slight demyelination before EGFR-TKI therapy; (A2) Brain MRI showed more ischemic foci in the frontal lobe, bilateral ventricle and basal ganglia region, lacunar infarction, demyelination, and encephalatrophy after EGFR-TKI therapy; (B) Chest CT with contrast of patient 4 showed partial response in chest lesions after EGFR-TKI treatment.

The patient was a 66-year-old female (Figure 3(Fig. 3)) who had a pre-existing depressive disorder and took Duloxetine regularly. In November 2015, this patient presented with cough and sputum. Chest CT scan and lung biopsy indicated lung adenocarcinoma with bilateral lung metastasis. Genetic testing showed that EGFR, EML4-ALK, and ROS-1 were negative. After two cycles of chemotherapy (taxol and cisplatin), the patient switched to other chemotherapy regiments (pemetrexed and nedaplatin) because of intolerable side effects (myelosuppression), and the treatment evaluation was partial remission. After eight cycles of chemotherapy, this patient received maintenance chemotherapy (pemetrexed). In June 2016, chest CT reexamination prompted disease progression, and the patient received other chemotherapy drugs (gemcitabine and carboplatin) and bevacizumab. In August 2016, the patient presented with headache. Chest CT reexamination showed disease progression. Cerebral MRI enhancement scanning showed some ischemic foci in the frontal lobe and slight demyelination. Repeating cerebrospinal fluid examination showed that the exfoliated cells were negative. After consultation with a neurologist, a neurosurgeon, and a radiologist, brain and meningeal metastases were not considered initially. After discussion and negotiation, the patient started targeted therapy (erlotinib, 150 mg, once a day). Periodic reviews suggested that some pulmonary tumor lesions were relieved, some lesions were stable, and clinical symptoms were significantly improved. In October 2017, the patient presented with headache again. Cerebral MRI enhancement scanning showed more ischemic foci in the frontal lobe, bilateral ventricle, and basal ganglia region; lacunar infarction; demyelination; and encephalatrophy. In addition, the patient exhibited obvious dysphoria. Cerebrospinal fluid examination was normal again, and the exfoliated cells in the cerebrospinal fluid were still negative. After consultation with a psychiatrist, anxiety and depression were considered. After pharmacological treatment, these symptoms did not improve, and the patient displayed paroxysmal of unconsciousness, incontinence of urine and feces, lack of communication and response, memory deterioration, and dim expression. The patient accepted cranial radiotherapy and Osimertinib. The headache started to alleviate, but the psychiatric symptoms of the patient still did not show substantial improvement.

### Case 5

**Figure 4:**
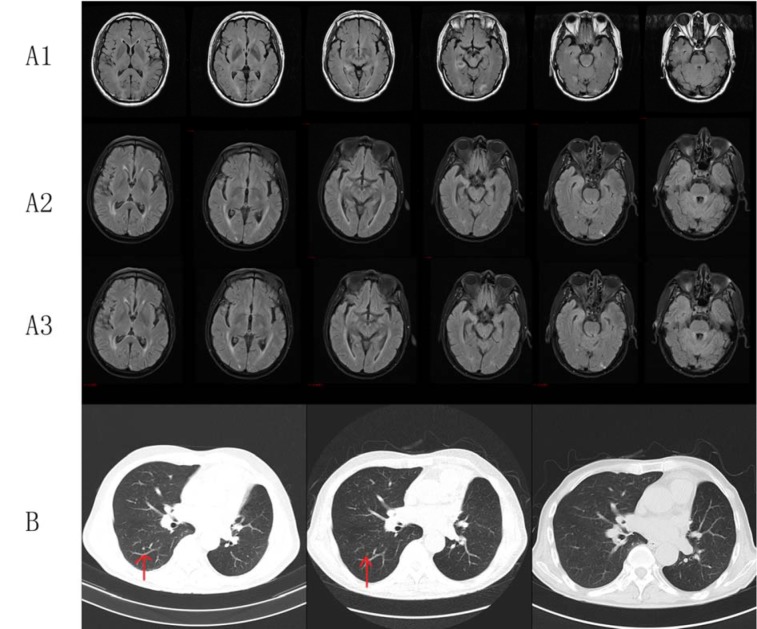
Clinical images of Case 5. (A1) Brain MRI showed multiple nodules in the brain, and metastatic tumor was considered; (A2) Brain MRI showed more ischemic foci and lacunar infarction in the bilateral frontal and parietal lobes and demyelination after 3 months of EGFR-TKI therapy; (A3) Brain MRI showed shrinking metastatic tumor in the brain, more and more ischemic foci and lacunar infarction in the bilateral frontal and parietal lobes, and demyelination after 6 months of EGFR-TKI therapy; (B) Chest CT with contrast of patient 5 showed the gradual disappearance of pulmonary nodules after EGFR-TKI treatment.

A 63-year-old male (Figure 4(Fig. 4)) initially presented with cough and sputum and was diagnosed with lung cancer. The patient received thoracoscope resection of the left lung upper lobe and lymph node dissection in December 2014. Postoperative pathology diagnosis demonstrated <upper lobe of left lung> invasive adenocarcinoma, (T2aN0M0 stage IB). After the surgery, the patient received four cycles of chemotherapy (taxol and nedaplatin). In April 2016, enhanced chest CT showed enlarged right-sided supraclavicular and mediastinal lymph node. In March 2017, the patient suffered from dizziness, headache, and unsteady gait. Cerebral MRI enhancement scanning showed multiple nodules in the brain. Metastatic tumor was considered. The patient received whole brain radiotherapy (WBRT) and then started targeted therapy (erlotinib, 150 mg, once a day) by himself. Reexamination indicated partial remission in the chest. After 5 months, the patient displayed balderdash, accompanied by glazed eyes and convulsions of the upper limbs. Cerebral MRI enhancement scanning at a local hospital showed severe encephalatrophy. The patient was treated with neurotrophic drugs, but he showed disturbance of consciousness and spontaneous recurrent seizures (drawn face, dull eyes, and muscular rigidity of both upper limbs). After 3 days, the patient died suddenly.

## Discussion

**Figure 5:**
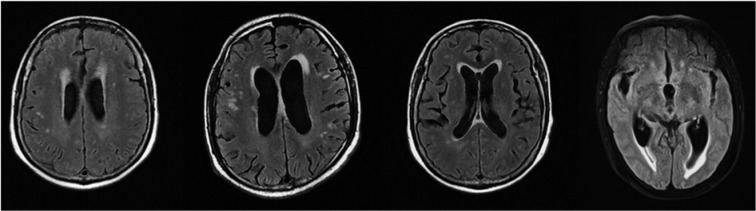
Representative MRI image of patients with ischemic foci and lacunar infarction in the brain after EGFR-TKI treatment

EGFR TKIs have revolutionized the treatment of EGFR mutant NSCLC. Compared with conventional chemotherapy agents, EGFR TKIs demonstrate a different adverse event (AE) profile, including hepatotoxicity, paronychia, diarrhea, stomatitis and mucositis, and cutaneous adverse events. However, the AE profile of a mental disorder or conscious disturbance remains lacking. This paper presented several patients with advanced lung adenocarcinoma who developed a mental disorder or conscious disturbance during EGFR-TKI treatment. The characteristics of these patients are as follows. 1) The main clinical symptoms of these patients were sluggishness, memory deterioration, cognitive disorder, and even hallucination. 2) For the pulmonary lesions (the tumor lesion in one patient was adrenal gland), the EGFR TKI therapy was effective for all four patients even though two patients were blind to eat drugs and two patients were EGFR negative by genetic testing. 3) Brain MRI revealed increased ischemic foci and lacunar infarction, serious encephalatrophy, and demyelination after EGFR-TKI therapy (Figure 5(Fig. 5)). 4) The interval time from EGFR-TKI therapy to onset of psychiatric symptoms was 3-12 months. 5) Except in 1 patient, no imaging or pathological evidence of brain or meningeal metastases was observed. 6) These psychiatric symptoms did not improve but worsened after taking antipsychotic drugs, suggesting that they were irreversible.

Basing on these features, we explored possible causes and summarized them as follows. First, paraneoplastic limbic encephalitis (PLE), a paraneoplastic neurological syndrome, should be considered. Because the limbic system is the primary component of memory, learning, and higher emotion, PLE is a rare disorder characterized by clinical presentations (e.g., cognitive disorders, hallucinations and other psychiatric syndromes, short-term memory loss, and seizures), presence of antineuronal antibodies, and imaging findings confirming involvement of the limbic system (Shen et al., 2018[18]). PLE has been most commonly associated with lung cancer, and 80 % of these cases are small cell lung cancer (Gozzard et al., 2015[7]). Brain MRI plays an important role in the diagnosis of limbic encephalitis. Anti-neural antibodies do not have very high sensitivity or specificity. PLE is a diagnosis of exclusion and confirmed by the pathological diagnosis of limbic encephalitis or mandatory components: clinical symptoms of PLE; a cancer diagnosis within 4 years of the onset of neuropsychiatric symptoms; exclusion of other neurologic causes; and evidence of limbic encephalopathy on MRI, electroencephalogram, anti-neural antibodies, or cerebrospinal fluid studies (Reck et al., 2014[17]; Ando et al., 2018[2]; Kuroda and Kawamura, 2015[12]; Lalani and Haq, 2012[13]). However, the five patients did not meet these criteria, especially the MRI imaging, which rendered these symptoms hard to explain by PLE. Second, brain or meningeal metastases need to be considered. Brain or meningeal metastases from solid tumors are associated with increased morbidity and mortality, which affect quality of life and survival. Patients can present with neurocognitive disorder, focal neurologic deficits, or symptoms such as headache, nausea and vomiting depending on the location, number, and size of the brain metastases (Aizer and Lee, 2018[1]; Berghoff and Preusser, 2018[4]; Bovi, 2018[6]). The diagnosis of brain metastases and meningeal metastases needs to combine neurologic symptoms, neuroimaging, and cerebrospinal fluid examination. However, among these five patients, only one patient considered brain metastases and received WBRT. Thus, whether patients with brain metastases or meningeal metastases can have negative imaging examination and cerebrospinal fluid examination needs to be considered. Last, EGFR is a tyrosine kinase that binds to many signaling proteins and stimulates the activation of many signaling pathways (Ayuso-Sacido et al., 2010[3]). It has three main downstream pathways: RAS/RAF/MAPK, PI3K/AKT, and JAK/STAT. These pathways stimulate mitosis, leading to cell proliferation and inhibition of apoptosis, which are crucial in normal cell growth (Wang, 2017[19]). EGFR signaling also affects many cellular events involved with corticogenesis, including neural cell proliferation, survival, differentiation, and migration (Zhu et al., 2000[20]). We assume that EGFR-TKI also affects the nerve cells in the progress of blocking the EGFR pathway. Thus, patients in EGFR-TKI treatment may have a mental disorder or conscious disturbance. The reason needs to be further explored.

Therefore, whether patients showing psychiatric symptoms have PLE or brain metastases or meningeal metastases in brain MRI and other examinations should be clarified. If the above factors are excluded, we need to consider the side effect of EGFR-TKIs. In consideration that these psychiatric symptoms could not improve after drug treatment, the best choice for these patients is to stop taking the drugs. 

## Conclusion

Here, we reported five cases of lung adenocarcinoma treated with EGFR-TKI that developed a mental disorder or conscious disturbance. This study suggests the need to consider neuropsychiatric symptoms in patients receiving EGFR-TKI treatment and explore the underlying mechanisms.
